# Spatial patterns of tau deposition are associated with amyloid, ApoE, sex, and cognitive decline in older adults

**DOI:** 10.1007/s00259-019-04669-x

**Published:** 2020-01-08

**Authors:** Joana B. Pereira, Theresa M. Harrison, Renaud La Joie, Suzanne L. Baker, William J. Jagust

**Affiliations:** 1 Division of Clinical Geriatrics, Department of Neurobiology, Care Sciences and Society, Karolinska Institute, Stockholm, Sweden; 2 Helen Wills Neuroscience Institute, University of California Berkeley, Berkeley, CA USA; 3 Memory and Aging Center, University of California, Oakland, CA USA; 4 Molecular Biophysics and Integrated Bioimaging, Lawrence Berkeley National Laboratory, Berkeley, CA USA; 5 Clinical Memory Research Unit, Department of Clinical Sciences, Lund University, Malmö, Sweden

**Keywords:** Flortaucipir PET, Older adults, Amyloid, APOE, Sex, Cognitive decline

## Abstract

**Purpose:**

The abnormal deposition of tau begins before the onset of clinical symptoms and seems to target specific brain networks. The aim of this study is to identify the spatial patterns of tau deposition in cognitively normal older adults and assess whether they are related to amyloid-β (Aβ), *APOE*, sex, and longitudinal cognitive decline.

**Methods:**

We included 114 older adults with cross-sectional flortaucipir (FTP) and Pittsburgh Compound-B PET in addition to longitudinal cognitive testing. A voxel-wise independent component analysis was applied to FTP images to identify the spatial patterns of tau deposition. We then assessed whether tau within these patterns differed by Aβ status, *APOE* genotype, and sex. Linear mixed effects models were built to test whether tau in each component predicted cognitive decline. Finally, we ordered the spatial components based on the frequency of high tau deposition to model tau spread.

**Results:**

We found 10 biologically plausible tau patterns in the whole sample. There was greater tau in medial temporal, occipital, and orbitofrontal components in Aβ-positive compared with Aβ-negative individuals; in the parahippocampal component in ε3ε3 compared with ε2ε3 carriers; and in temporo-parietal and anterior frontal components in women compared with men. Higher tau in temporal and frontal components predicted longitudinal cognitive decline in memory and executive functions, respectively. Tau deposition was most frequently observed in medial temporal and ventral cortical areas, followed by lateral and primary areas.

**Conclusions:**

These findings suggest that the spatial patterns of tau in asymptomatic individuals are clinically meaningful and are associated with Aβ, *APOE* ε2ε3, sex and cognitive decline. These patterns could be used to predict the regional spread of tau and perform in vivo tau staging in older adults.

**Electronic supplementary material:**

The online version of this article (10.1007/s00259-019-04669-x) contains supplementary material, which is available to authorized users.

## Background

Alzheimer’s disease (AD) is characterized by a long-lasting preclinical phase, in which the presence of progressively increasing pathology is not yet accompanied by clinical symptoms [1]. In this preclinical phase, biomarkers of tau pathology have important prognostic value and could be used to predict cognitive decline and identify individuals at higher risk of developing AD [2]. Thanks to the recent development of tau PET tracers, it is now possible to measure the regional distribution and topological patterns of tau, and assess how they are related to other biomarkers of early AD [3]. However, so far, most tau PET studies in older adults have focused on a single brain area or independent regions of interest, despite growing evidence that tau deposits in different areas are not independent from each other but are strongly correlated, forming spatial patterns or covariance networks [4–8].

The relationship between the spatial patterns of tau and biomarkers associated with risk of developing AD remain unexplored in older adults. In particular, the effects of Aβ-positivity, *APOE*, sex, and cognition on these patterns have not yet been assessed. In addition, no studies have examined tau PET differences between *APOE* ε2ε3 and ε3ε3 carriers [9, 10]. In this study, we sought to address these issues by identifying the spatial patterns of tau in older adults and assessing whether tau in these patterns is related to amyloid, *APOE*, sex, or longitudinal cognitive decline. We also sorted the spatial patterns based on the frequency of tau positivity across subjects to determine a proxy for regional tau spread.

## Methods

### Participants

One hundred and fourteen cognitively normal individuals ≥ 60 years of age were included from the Berkeley Aging Cohort Study. All individuals underwent structural MRI, Pittsburgh Compound-B PET (PiB), and Flortaucipir (FTP) PET imaging in addition to neuropsychological testing. Inclusion criteria included a baseline Mini-Mental State Examination (MMSE) score ≥ 25; normal performance on cognitive tests; no neurological, psychiatric, or major medical illness; no medications affecting cognition; and no imaging contraindications, and that all participants were community-dwelling.

### Neuropsychological test battery

Eighty-six of the 114 subjects underwent longitudinal neuropsychological testing before and up to 1 year after the date of the FTP scan. These subjects had between 2 and 11 cognitive testing sessions (mean, 3.8 ± 2.4) over a period of 4.6 ± 2.9 years, with an average delay of 1.2 ± 0.4 years between sessions. For each testing session, composite *Z* scores for memory and executive function domains were created, as previously described [11]. These *Z* scores were calculated using the means and standard deviations from the first cognitive session data of a larger sample of 150 healthy participants from the Berkeley Aging Cohort Study that also included the 86 older adults studied here. The memory composite score included the short- and long-delay free recall tests of the California Verbal Learning Test [12] and the Visual Reproduction test of the Wechsler Memory scale [13]. The executive function composite score included the Digit-Symbol test [14], Trail Making Test B minus A [15], and Stroop number of correct words in 1 min [16].

### APOE genotyping

The determination of *APOE* alleles was performed using a TaqMan Allelic Discrimination Assay using a Real-Time PCR system (Applied Biosystems, Foster City, CA), as previously described [17]. One subject had missing *APOE* information.

### Image acquisition

All PET scans were acquired at the Lawrence Berkeley National Laboratory on a Siemens (Erlangen, Germany) Biograph 6 Truepoint PET/computed tomography (CT) scanner in 3D acquisition mode. FTP was synthesized using a TracerLab FXN-Pro (GE Medical Systems, Milwaukee, WI) synthesis module with a modified protocol based on an Avid Radiopharmaceuticals (Philadelphia, PA) protocol. Participants were injected with 10 mCi of tracer and scanned in list mode from 75 to 105 min post-injection. Data was subsequently reconstructed as 4 × 5-min frames within 80 to 100 min post-injection. PiB was also synthesized according to a previously published protocol [18]. Immediately after the intravenous injection of approximately 15 mCi of PiB, 90 min of dynamic acquisition frames were obtained (4 × 15, 8 × 30, 9 × 60, 2 × 180, 10 × 300, and 2 × 600 s). Both FTP and PiB images were reconstructed using an ordered subset expectation maximization algorithm with weighted attenuation and smoothed with a 4-mm Gaussian kernel with scatter correction (image resolution = 6.5 × 6.5 × 7.25 mm^3^).

T1-weighted structural MRI images were acquired on a 1.5-T Siemens Magnetom.

Avanto scanner using a magnetization-prepared rapid gradient echo sequences with the following parameters: repetition time = 2110 ms, echo time = 3.58 ms, flip angle = 15°, voxel size = 1mm isotropic.

### Image preprocessing

Both PiB and FTP PET frames were motion-corrected, time-averaged, and coregistered to their T1-weighted images. For PiB images, distribution volume ratios (DVRs) were calculated using Logan graphical analysis on PiB frames corresponding to 35 to 90 min post-injection using all the frontal, temporal, and parietal regions provided by the Desikan-Killiany atlas of FreeSurfer as well as a cerebellar gray matter reference region [19, 20]. Subjects were classified as Aβ-positive based on a mean global cortical DVR > 1.065 [21, 22].

For FTP, standardized uptake value ratio (SUVR) images were created using the mean inferior cerebellar gray matter uptake as the reference region [23–25]. These images were warped to MNI152 space using FSL (https://fsl.fmrib.ox.ac.uk/fsl/fslwiki) and smoothed using an 8-mm Gaussian filter. Warping to MNI was performed using the transformation parameters derived from warping the T1-weighted images to the MNI152 template. The average delay between the PiB and FTP scans was 24.8 ± 99.2 days.

In this study, we did not apply partial volume corrections (PVC) to the FTP data because methods suitable for voxel-wise analyses fail to account for tracer binding in the multiple brain compartments (CSF, white matter, extracranial uptake) that can affect FTP images.

### Tau PET independent component analysis

FTP PET images were submitted to an independent component analysis using the group ICA of functional MRI Toolbox (GIFT) [26]. This data-driven analysis decomposes the data into independent components or spatial patterns of correlated signals from distributed brain areas across subjects. To determine the optimal number of components, we used the minimum description length criterion [27]. In addition, in order to produce reliable components, we used the ICASSO algorithm implemented in GIFT, which ran ICA 10 times before selecting stable components that corresponded to tight clusters. A brain mask that excluded the basal ganglia, brainstem, cerebellum, subcortical white matter, and cerebrospinal fluid (including the choroid plexus) was included in the analyses to minimize contamination from regions considered to represent off-target binding in FTP data [28–30]. After identifying the spatial patterns, we thresholded them with a *Z* score > 2.0, which corresponds to a two-tailed significance value of *p* < 0.05.

### Statistical analyses

Differences in baseline characteristics between groups were analyzed using chi-squared tests for binary variables and analyses of variance for continuous variables. To assess whether tau burden within each spatial component differed by Aβ status, *APOE* genotype and sex, we compared mean SUVRs within these components between Aβ− and Aβ+ groups, subjects with different *APOE* genotypes, and male and female participants. These analyses were carried out using permutation tests with 1000 replicates, while controlling for age, sex, education, and PiB DVR, when appropriate. To identify and exclude potential outliers in tau SUVRs, we applied the Tukey method (1.5 × interquartile range). Adjustment for multiple comparisons was performed using false discovery rate (FDR) corrections [31] at *q* < 0.05. For all significant group differences, we calculated effect sizes using Cohen’s *d* [32].

To evaluate whether the spatial patterns of tau were associated with change in cognition over time, we fitted linear mixed effect models, implemented in R using “lme4.” In these models, we included memory or executive scores as dependent variables, and tau component SUVRs, time since first cognitive evaluation, age, sex, education, and PiB DVR as fixed effects. These models included all main effects, the interaction between tau SUVRs and time, and random effects for intercepts and slopes: cognitive scores ~ time × tau network SUVR + age + sex + education + PIB DVR + (time|subject). We built separate models for each tau component.

Finally, to determine the order of tau spreading across the different components, we selected Aβ− individuals whose FTP SUVR values in the entorhinal cortex were < 1.2 to obtain regional mean and standard deviation values of SUVR, following a previously described procedure [33]. Using these means and standard deviations, we computed *Z* scores for the mean SUVR in each tau component across all participants. For each participant, components with a *Z* score > 2.5 were considered to be positive for tau deposition. Using this approach, the frequency of positive tau deposition was assessed for each component. Components were then ordered from highest to lowest frequency since we assumed that components with a higher frequency of positive tau deposition comprised earlier accumulating regions. This approach allowed us to estimate spatial tau spread using cross-sectional data.

## Results

### Participants

Characteristics of the sample are summarized in Table 1. The majority of participants had one of the following *APOE* genotypes: ε2ε3 (*n* = 11), ε3ε3 (*n* = 71), or ε3ε4 (*n* = 28). In addition, 3 participants were ε2ε4 carriers, which were excluded from the analyses due to the small number of subjects in this group.

**Table 1:** Characteristics of the sample

	All subjects (*n* = 114)	Aβ− (*n* = 67)	Aβ+ (*n* = 47)	ApoE ε2/ε3 (*n* = 11)	ApoE ε3/ε3 (*n* = 71)	ApoE ε3/ε4 (*n* = 28)	Men (*n* = 45)	Women (*n* = 69)
Age (years)	76.4 (6.3)	76.3 (7.4)	76.6 (4.3)	75.2 (7.6)	76.4 (6.8)	76.6 (4.6)	77.1 (6.7)	75.9 (6.0)
Sex (m/f)	45/69	27/40	18/29	7/4	25/46	12/16	-	-
Education (years)^a,b^	16.8 (1.8)	17.2 (1.8)	16.4 (1.8)	16.3 (0.6)	17.2 (1.7)	16.4 (2.3)	17.1 (1.6)	16.7 (2.0)
PiB DVR^a,b,c,d^	1.2 (0.2)	1.02 (0.0)	1.34 (0.3)	1.02 (0.04)	1.09 (0.16)	1.30 (0.3)	1.12 (0.2)	1.17 (0.2)
APOE (e2/e3/e4)^a^	11/71/28	10/52/5	1/19/23	-	-	-	7/25/12	4/46/16
MMSE	28.8 (1.2)	28.9 (1.0)	28.5 (1.4)	29.2 (0.8)	28.8 (1.2)	28.5 (1.3)	28.8 (1.2)	28.7 (1.2)
Memory	0.02 (0.9)	0.14 (0.9)	− 0.15 (0.9)	0.54 (0.9)	− 0.04 (0.9)	0.10 (0.8)	− 0.08 (0.8)	0.09 (1.0)
Executive function^e^	0.06 (0.7)	0.11 (0.7)	− 0.01 (0.7)	0.17 (0.9)	0.01 (0.7)	0.22 (0.7)	− 0.12 (0.6)	0.18 (0.8)

Values in the table correspond to means followed by (standard deviation), except for sex and APOE, which correspond to number of subjects in each category

^a^Significant differences between Aβ− and Aβ+ groups (*p* < 0.05)

^b^Significant differences between ApoE ε2/ε3 and ApoE ε3/ε3 groups (*p* < 0.05)

^c^Significant differences between ApoE ε2/ε3 and ApoE ε3/ε4 groups (*p* < 0.05)

^d^Significant differences between ApoE ε3/ε3 and ApoE ε3/ε4 groups (*p* < 0.05)

^e^Significant differences between men and women (*p* < 0.05)

In this study, Aβ-positive individuals were less educated (*d* = 0.4, *p* = 0.031) and had a higher prevalence of the *APOE* ε3ε4 genotype (*d* = 1.1, *p* < 0.001) than their Aβ-negative peers. As expected, ε3ε4 carriers showed higher PiB DVRs compared with both ε2ε3 (*d* = 1.4, *p* = 0.004) and ε3ε3 (*d* = 0.9, *p* < 0.001) carriers, and ε2ε3 were less educated than ε3ε3 carriers (*d* = 0.7, *p* = 0.003). In our sample, men had lower executive function scores compared with women (*d* = 0.5, *p* = 0.025).

### Spatial patterns of tau deposition in older adults

The optimal number of components identified in FTP data was 15, which explained 85.2% of the total variance. Five of these components were excluded from the analyses since they had white matter voxels or consisted of thin clusters surrounding the meninges, which is an off-target binding region for ^18^F-FTP data. The excluded components can be found in Supplementary Fig. 1. After excluding the noisy components, the total variance explained by the data was 48.5%. This was due to the fact that one of the excluded components explained a high proportion of the variance or a wide spread in the FTP values in our dataset (22.9%). This component was characterized by high tau signal in a thin elongated cluster surrounding the meninges (component 4 in Supplementary Fig. 1). The meninges have been previously described as an off-target binding area for FTP as well as other tau PET tracers. Importantly, their signal varies across subjects and can potentially bleed into cortical areas [34, 35]. Our independent component analyses were able to identify this source of variable off-target binding, which allowed us to effectively remove it from our main analyses. The variance explained by each of the 15 components can be found in Supplementary Table 1.

**Fig. 1:**
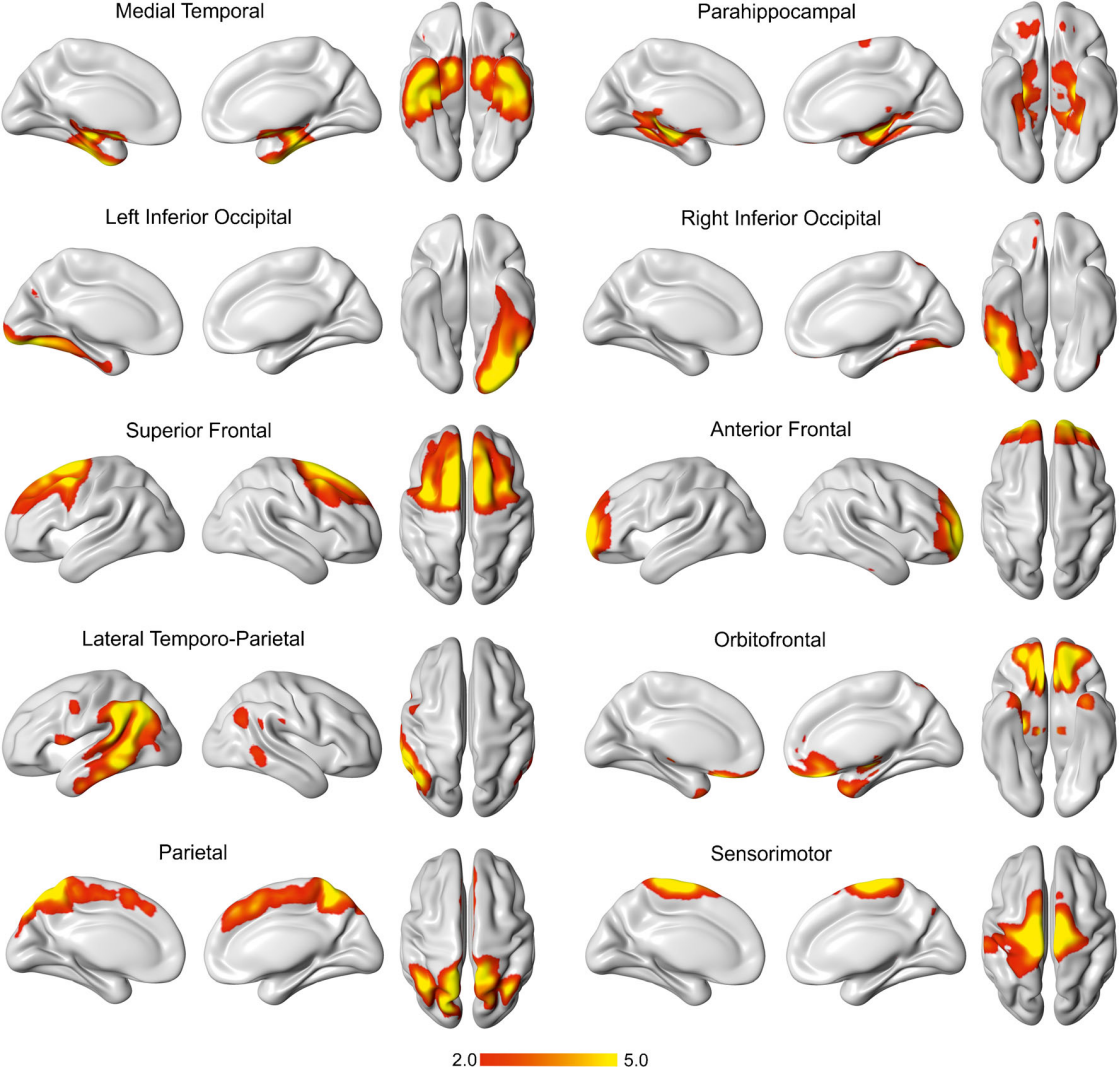
Spatial patterns of tau deposition in aging. We identified 10 independent components for tau in the analyses of FTP data. These components were thresholded with a *Z* score > 2.0

After excluding the noisy components, the remaining components (*n* = 10) were included in further analyses. These components involved bilateral gray matter regions, except for 2, which were unilateral (Fig. 1). The bilateral components included medial temporal, parahippocampal, superior and anterior frontal, lateral temporo-occipital, orbitofrontal, parietal, and sensorimotor areas. All of these components were symmetric, with the exception of the lateral temporo-parietal component, which showed greater involvement on the left hemisphere. The unilateral components included left and right inferior occipital areas. The distribution of tau SUVRs in these components can be found in Supplementary Fig. 2.

### Tau burden in spatial patterns differs by Aβ status, *APOE* genotype and sex

**Fig. 2:**
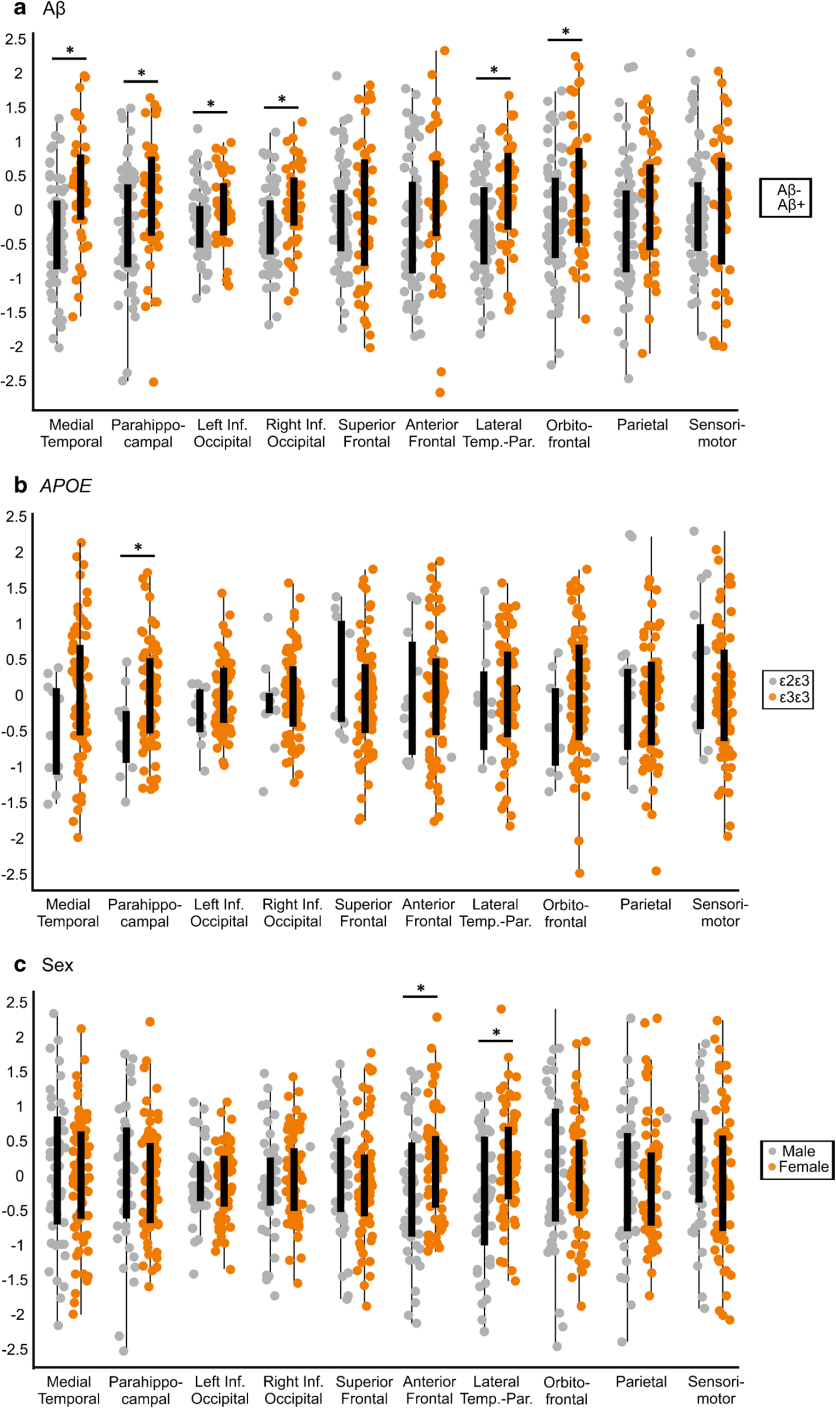
Tau burden within spatial patterns differs by Aβ, *APOE*, and sex in older adults. We found significantly higher tau burden in some of the spatial patterns in Aβ-positive compared with Aβ-negative peers, *APOE* ε3ε3 compared with ε2ε3 carriers, and in women compared with men. Asterisk indicates significant differences between groups, after regressing out the effects of age, sex, education for Aβ groups; age, sex, education, and PiB DVR for *APOE* groups; and age, education, and PiB DVR for sex groups. All results were corrected for multiple comparisons using FDR (*q* < 0.05)

Our group analyses showed that Aβ-positive individuals had higher tau SUVRs within the medial temporal (*d* = 0.8, *p* < 0.001), parahippocampal (*d* = 0.5, *p* = 0.008), left and right inferior occipital (*d* = 0.5, *p* = 0.005; *d* = 0.5, *p* = 0.003), lateral temporo-parietal (*d* = 0.5, *p* = 0.001) and orbitofrontal (*d* = 0.5, *p* = 0.008) components compared with Aβ-negative subjects (Fig. 2a), after FDR corrections.

In addition, we found higher tau SUVRs in the parahippocampal component (*d* = 0.7, *p* = 0.008) in ε3ε3 carriers compared with those in ε2ε3 carriers after adjusting for PiB DVR and for multiple comparisons (Fig. 2b). When PiB was excluded as a covariate from these analyses, we observed tau increases in additional networks in ε3ε3 and ε3ε4 compared with ε2ε3 carriers (Supplementary Table 2).

Finally, our analyses comparing male and female participants showed that women had higher tau in the anterior frontal (*d* = 0.5, *p* = 0.007) and lateral temporo-parietal (*d* = 0.6, *p* < 0.001) components compared with men (Fig. 2c) after adjusting for PiB and FDR. These differences were still significant after excluding PiB as a covariate (Supplementary Table 2).

### Spatial tau patterns are associated with longitudinal cognitive decline

Our mixed effects models did not show any significant interactions between time and the different tau patterns in cognitive decline after adjusting for multiple comparisons with FDR (*p* < 0.05). However, at an uncorrected level, we identified an interaction between time and the parahippocampal (estimate, − 0.15; SD, 0.05; *t* score, − 2.891, *p* = 0.007), left inferior occipital (estimate, − 0.20; SD, 0.08; *t* score, − 2.613, *p* = 0.010), and lateral temporo-parietal (estimate, − 0.17; SD, 0.08; *t* score, − 2.201, *p* = 0.032) components (Supplementary Table 3), indicating that participants with higher tau within these patterns had worse longitudinal decline in memory. In addition, we also found an interaction between the superior frontal tau pattern and time (estimate, − 0.13; SD, 0.06; *t* score, − 2.305, *p* = 0.024) (Supplementary Table 3), indicating that participants with higher tau within this pattern had worse executive decline.

We also ran additional analyses using linear mixed effects models that included interactions between time and all covariates (age, sex, education, PIB DVR). These models showed lower AIC and BIC values than the models with a single interaction term between time and tau (Supplementary Table 4), indicating they were a worse fit to our data.

### In vivo staging of tau spatial patterns

**Fig. 3:**
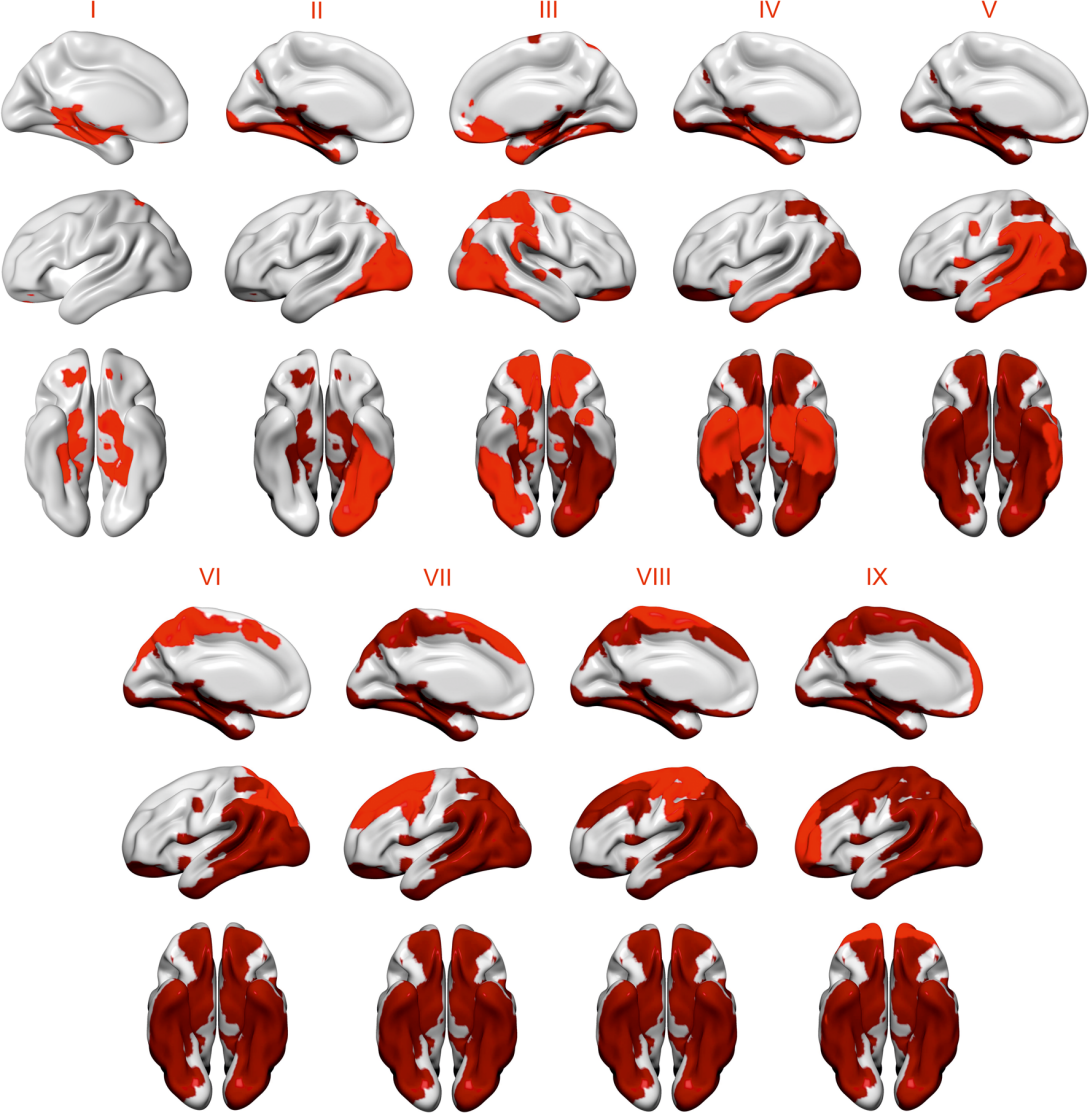
Progression of tau pathology across different spatial patterns. We used the means and standard deviations of Aβ− individuals with entorhinal tau < 1.2 to identify individuals with high tau in the different spatial patterns. The patterns that were determined to show increased binding in a greater number of participants were considered to be regions of early tau deposition. Using this approach, we found a spreading order of tau across the spatial patterns from limbic and ventral areas, to lateral and medial posterior regions, and frontal and sensorimotor cortices. Patterns colored in light red correspond to a newly added stage, whereas patterns colored in dark red correspond to previous stage

The progression of tau pathology across the spatial patterns is displayed in Fig. 3, and a table of overlaps of high tau across the patterns in different subjects is provided in Supplementary Table 5. In the whole cohort, we found that tau deposition was most frequently observed in the parahippocampal (25.4%, *n* = 29) pattern (I). This was followed by regional involvement of ventral areas that included the left inferior occipital (15.8%, *n* = 18) (II), right inferior occipital (12.3%, *n* = 14), orbitofrontal (12.3%, *n* = 14) (III), and medial temporal (11.4%, *n* = 13) (IV) components. Once most of the ventral areas were affected by tau pathology, the more frequent set of regions with high tau deposition included lateral and medial cortical areas such as the lateral temporo-parietal (6.1%, *n* = 7) (V), parietal (5.3%, *n* = 6) (VI), and superior frontal (3.5%, *n* = 4) (VII) components. Finally, the least affected patterns with tau were the sensorimotor (2.6%, *n* = 3) (VIII) and anterior frontal (1.8%, *n* = 2) (IX) components.

## Discussion

With upcoming therapeutic approaches based on tau pathology, new tau biomarkers are urgently needed for early AD [36]. In this study, we show that the spatial patterns of tau deposition could potentially be used as early biomarkers of AD and detect changes in clinically normal older individuals who are Aβ-positive, *APOE* ε3ε3 carriers, or women. In addition, the association we found between some of these patterns and cognitive decline suggest they could also be used to understand the variability in longitudinal cognitive trajectories among older adults.

There is increasing evidence showing that, rather than appearing in unrelated brain regions, tau pathology occurs within the boundaries of distributed networks [37, 38]. However, these networks have been poorly characterized in cognitively normal individuals, with only one study identifying four clusters of areas with tau deposition [8]. Our data-driven approach detected 10 independent spatial patterns of tau across temporal (medial temporal, parahippocampal, lateral temporo-parietal), occipital (left and right inferior occipital), frontal (orbitofrontal, middle frontal, anterior frontal), parietal, and sensorimotor regions. This finding might seem surprising, as several studies have shown that tau is either absent or restricted to temporal regions in cognitively normal individuals [39–41]. Our study shows that an unsupervised network approach may reveal additional patterns in tau PET data, which can be used to understand why some individuals, despite being clinically normal, are more prone to accumulate tau in specific brain areas. For instance, it has been previously shown that Aβ-positive individuals show higher tau deposition than their Aβ-negative counterparts [40–42] due to some interaction between amyloid and tau that is not fully understood [42]. Here, we show that this relationship can be observed not only in temporal areas but also in inferior occipital, lateral temporo-parietal, and orbitofrontal regions, confirming that Aβ may accelerate the spread of tau deposition outside the temporal cortex into adjacent areas [42]. In addition to Aβ, the role of sex in tau deposition has been recently assessed in clinically normal adults, with women showing greater temporal tau than men in the presence of high Aβ burden [43]. In our study, we found that women had greater tau in temporo-parietal and anterior frontal components compared with men, indicating that the effects of sex on tau deposition might be more widespread than previously thought. The higher AD prevalence previously reported in women [44] together with evidence of heightened female inflammatory responses [45] and relationship between tauopathy and menopause [46] could potentially explain why women are more vulnerable to tau deposition than men. Future studies are needed to determine the exact biological underpinnings of sex differences in tau deposition. Finally, to our knowledge, our study is the first showing the protective value of the *APOE* ε2 allele against in vivo tau deposition in clinically normal adults. We found that the ε2ε3 genotype was associated with lower tau in parahippocampal areas compared with the more frequent ε3ε3 genotype, before and after controlling for Aβ. In contrast, we found that the increases of tau load in ε3ε4 carriers were mostly mediated by Aβ. Our results agree with previous findings of reduced neurofibrillary tangle pathology in ε2 carriers [47], and the well-known associations between amyloid *APOE* ε4 and amyloid [48], which are not independent from each other.

One of the earliest cognitive changes in aging, and the one that has probably received most attention due to its association with AD, is the decline of memory. Here we found that higher tau load in the parahippocampal, left inferior occipital, and lateral temporo-parietal patterns predicted retrospective longitudinal decline in memory in older adults, although these associations did not remain significant after adjustment for multiple comparisons. These results potentially strengthen the hypothesis that tau pathology plays a key role in memory decline in aging [49], which is also strongly related to dysfunction of temporal and some parietal regions. In addition, we found that higher tau in the superior frontal pattern predicted retrospective decline in executive functions, a finding that has not yet been reported by previous studies. There is mounting evidence showing that the decline of executive functions is associated with dysfunction in frontal brain areas in aging [50]. Our findings provide evidence that this relationship might be at least partially mediated by tau and explain some of the variability observed in executive abilities in older adults [51]. However, this association did not survive FDR corrections and thus should be interpreted with caution.

In this study, to determine the “spread” of tau across the different spatial components, we calculated the frequency of high tau in each component in all participants. This approach showed that tau deposition was most frequent in the parahippocampus, followed by ventral areas such as the inferior temporal, inferior occipital, and orbitofrontal cortices. Then, from these ventral areas, tau spread into lateral temporo-parietal and medial parietal regions, and finally reached frontal areas and the sensorimotor cortex. This regional progression of tau largely mirrors the spread of neurofibrillary tangles previously reported by Braak et al. [52]. Interestingly, by using spatial patterns of tau deposition, we were able to reproduce the spread of tau from transentorhinal areas to other ventral areas of the brain [52], suggesting that the spatial information of our components might be useful for in vivo staging of tau pathology. Our findings also largely agree with those reported by a previous study specifically assessing the regional spread of tau across regions of interest [33].

Our study has some limitations. Although comparable with previous tau PET studies, our sample size was modest and might have limited our ability to detect additional spatial patterns or their value in characterizing Aβ, *APOE*, and sex effects. In particular, the number of ε2ε3 carriers was very small (*n* = 11), in agreement with the low prevalence of this genotype in the population [53]. Thus, our findings should be considered preliminary and replicated in future studies. In addition, not all individuals had the same number of longitudinal cognitive visits, something we dealt with using linear mixed effects models. Moreover, although we did our best to reduce partial volume effects by excluding regions known to have off-target binding in FTP data such as the basal ganglia, the choroid plexus, and the meninges [28–30], it is still possible that off-target binding could have added some noise to our analyses. Our data-driven approach based on independent component analyses has been previously described as a useful technique to separate signal from noise [54]. In fact, in our study, it was able to detect noisy components in thin areas surrounding the meninges and in the white matter, which were excluded from the analyses. In particular, the component surrounding the meninges explained a high percentage of the variance in our dataset (22.9%), in line with previous evidence showing that several tau PET tracers bind strongly to the meninges [34, 35, 55], which tends to spill into adjacent cortical areas. The reason for this strong off-target binding in the meninges and adjacent areas is unknown but a previous study showed that it seems to be more frequent in individuals who do not exhibit specific binding in regions typically associated with neurofibrillary tangles [55] such as cognitively normal subjects without amyloid pathology, who constituted more than 50% of our sample. Thus, we hope that, by applying an independent component analysis to FTP data, we removed as much noise as possible from our analyses. Finally, the analyses involving cognition did not survive control for multiple comparisons probably due to the fact that the subjects of our sample were cognitive normal and did not show strong memory or executive decline over time. Future studies are needed that allow assessing the relationship between the tau patterns and longitudinal cognitive trajectories for several decades, during which greater cognitive changes are more likely to occur.

## Conclusions

Our findings indicate that distinct spatial tau patterns are present in cognitively normal adults and Aβ-negativity, ε2ε3 carriership, and male sex are protective factors against tau deposition within these patterns. In light of the increasing recognition of AD as a network disorder that affects distributed brain systems [55], we hope that our findings might encourage future studies to analyze tau PET data using multivariate approaches that can characterize tau pathology across brain networks.

## Electronic supplementary material

ESM 1 (DOCX 20675 kb)
