# Supplementary material for: Spatial patterns of tau deposition are associated with amyloid, ApoE, sex, and cognitive decline in older adults
Source: Eur J Nucl Med Mol Imaging. 2020 Jan 8;47(9):2155–64. doi: 10.1007/s00259-019-04669-x (PMC7338820; doi:10.1007/s00259-019-04669-x)
Supplement: Supplementary file 1 — (DOCX 20675 kb) [file 259_2019_4669_MOESM1_ESM.docx]

**Supplemental Figure 1.** **Excluded tau components**


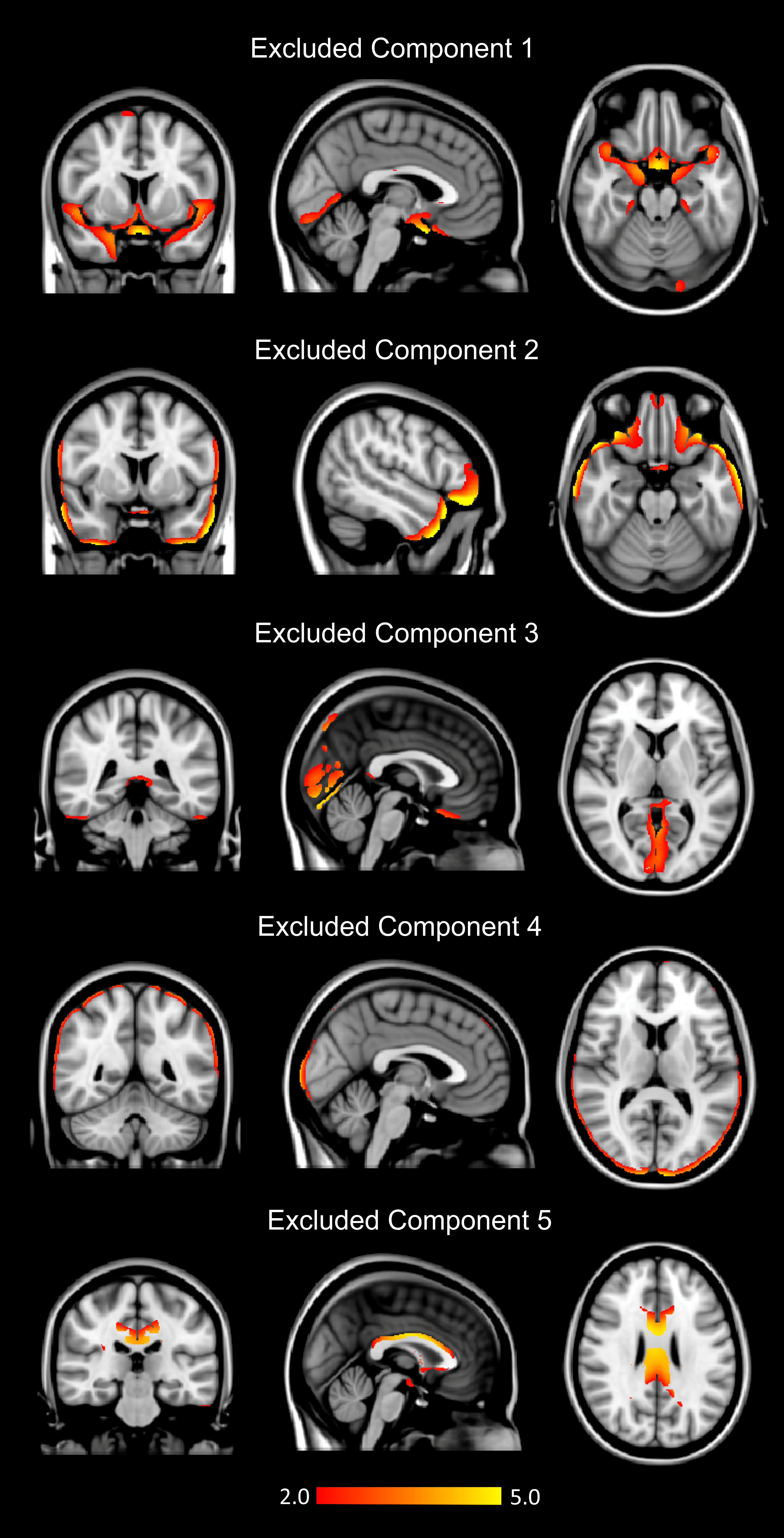


Five tau components were excluded from the analyses since they included white matter voxels or consisted of thin clusters surrounding the meninges, which is an off-target region for FTP PET. The excluded components are shown above threshold with z > 2.0.

**Supplemental Figure 2.** **Distribution of tau SUVRs within each pattern**

**

**

Kernel density plots of tau SUVRs in each of the main 10 components.

**Supplementary Table 1. Variance explained by each tau component extracted from the independent component analysis**

|  | **Variance** |
| --- | --- |
| **Medial Temporal** | 1.9% |
| **Parahippocampal** | 3.4% |
| **Left Inf. Occipital** | 3.6% |
| **Right Inf. Occipital** | 1.3% |
| **Superior Frontal** | 13.2% |
| **Anterior Frontal** | 9.3% |
| **Lat. Temporo-Parietal** | 1.9% |
| **Orbitofrontal** | 3.6% |
| **Parietal** | 3.2% |
| **Sensorimotor** | 7.0% |
| **Noise 1** | 1.8% |
| **Noise 2** | 1.1% |
| **Noise 3** | 6.8% |
| **Noise 4** | 22.9% |
| **Noise 5** | 4.1% |

**Supplementary Table 2. Differences in tau network SUVRs between APOE and sex groups without adjusting for PIB DVR**

| **Tau**  **networks** | **ApoE ε2/ε3**  **(n = 11)** | **ApoE ε3/ε3**  **(n = 71)** | **ApoE ε3/ε4**  **(n = 28)** | **Men**  **(n = 45)** | **Women**  **(n = 69)** | **ApoE ε2/ε3 vs**  **ApoE ε3/ε3**  **(p value)** | **ApoE ε2/ε3 vs**  **ApoE ε3/ε4**  **(p value)** | **ApoE ε3/ε3 vs**  **ApoE ε3/ε4**  **(p value)** | **Men**  **vs**  **Women**  **(p value)** |
| --- | --- | --- | --- | --- | --- | --- | --- | --- | --- |
| **Medial temporal** | -0.45 (0.39) | -0.08 (0.50) | -0.02 (0.54) | -0.10 (0.56) | -0.10 (0.48) | **0.009** | **<0.001** | 0.037 | 0.382 |
| **Parahippocampal** | -0.11 (0.87) | -0.08 (0.96) | 0.08 (10.00) | -0.36 (10.06) | 0.19  (0.79) | **0.009** | **<0.001** | 0.027 | 0.169 |
| **Left inferior occipital** | -0.19 (0.67) | -0.07 (0.92) | 0.01  (0.85) | -0.01 (0.97) | -0.12 (0.84) | **0.008** | 0.012 | 0.319 | 0.502 |
| **Right inferior occipital** | -0.73 (0.65) | -0.11 (0.83) | 0.23  (0.81) | -0.10 (0.88) | -0.04 (0.85) | 0.189 | 0.161 | 0.269 | 0.098 |
| **Superior frontal** | 0.14 (0.77) | 0.00  (0.80) | -0.20 (10.02) | -0.03 (0.85) | -0.06 (0.88) | 0.298 | 0.172 | 0.163 | 0.466 |
| **Anterior frontal** | -0.31 (0.67) | -0.12 (0.76) | 0.03  (0.95) | -0.34 (0.78) | 0.14  (0.71) | 0.462 | 0.294 | 0.234 | **0.001** |
| **Lateral Temporo-Parietal** | -0.65 (0.57) | -0.08 (0.78) | 0.28  (0.79) | 0.06  (0.84) | -0.08 (0.73) | 0.206 | 0.164 | 0.190 | **<0.001** |
| **Orbitofrontal** | 0.27 (0.93) | 0.04  (0.91) | -0.09 (10.01) | 0.15  (0.89) | -0.05 (0.98) | 0.054 | **0.009** | 0.055 | 0.467 |
| **Parietal** | -0.45 (0.63) | -0.02 (0.83) | 0.32 (10.09) | 0.04 (10.10) | 0.02  (0.85) | 0.347 | 0.239 | 0.316 | 0.281 |
| **Sensorimotor** | -0.28 (0.58) | -0.11 (0.58) | -0.03 (0.72) | -0.20 (0.68) | -0.04  (0.64) | 0.209 | 0.140 | 0.268 | 0.146 |

Mean tau SUVRs for the different networks after regressing out the effects of age, gender and education (for APOE group comparisons) or age and education (for sex group comparisons). Differences between groups were calculated using 1000 permutation tests. P values in bold indicate significant group differences after adjusting for multiple comparisons with FDR (q < 0.05).

**Supplementary Table 3. Main effects of each tau component and interaction between tau components with time in longitudinal analyses of memory and executive cognitive changes**

|  | **Memory scores ~ time * tau network SUVR + age + sex + education + PIB DVR + (time\|subject)** | | | **Executive scores ~ time * tau network SUVR + age + sex + education + PIB DVR**  **+ (time\|subject)** | | |
| --- | --- | --- | --- | --- | --- | --- |
|  | **Estimate (SD)** | **t score** | **P value** | **Estimate (SD)** | **t score** | **P value** |
| **Medial temporal**  ***Main effect***  ***Interaction × time*** | -1.18 (0.76)  -0.14 (0.07) | -1.556  -1.991 | 0.124  0.054 | -0.05 (0.50)  -0.03 (0.06) | -0.095  -0.503 | 0.924  0.618 |
| **Parahippocampal**  ***Main effect***  ***Interaction × time*** | -0.03 (0.56)  -0.15 (0.05) | -0.049  -2.891 | 0.961  0.007* | -0.03 (0.37)  0.01 (0.05) | -0.082  0.308 | 0.935  0.760 |
| **Left inferior occipital**  ***Main effect***  ***Interaction × time*** | -0.29 (0.49)  -0.20 (0.08) | -0.587  -2.613 | 0.559  0.010* | -0.09 (0.32)  -0.07 (0.06) | -0.276  -1.110 | 0.784  0.270 |
| **Right inferior occipital**  ***Main effect***  ***Interaction × time*** | -1.05 (0.88)  -0.16 (0.10) | -1.198  -1.662 | 0.235  0.104 | -0.19 (0.58)  -0.05 (0.08) | -0.327  -0.646 | 0.744  0.521 |
| **Superior frontal**  ***Main effect***  ***Interaction × time*** | -0.35 (0.52)  -0.12 (0.07) | -0.673  -1.599 | 0.503  0.114 | -0.07 (0.34)  -0.13 (0.06) | -0.198  -2.305 | 0.843  0.024* |
| **Anterior frontal**  ***Main effect***  ***Interaction × time*** | 0.40 (0.61)  0.04 (0.06) | 0.648  0.576 | 0.519  0.567 | 0.43 (0.39)  -0.03 (0.05) | 1.078  -0.664 | 0.285  0.510 |
| **Lateral Temporo-Parietal**  ***Main effect***  ***Interaction × time*** | -0.74 (0.68)  -0.17 (0.08) | -1.084  -2.201 | 0.282  0.032* | -0.15 (0.45)  -0.05 (0.06) | -0.326  -0.836 | 0.745  0.406 |
| **Orbitofrontal**  ***Main effect***  ***Interaction × time*** | 0.29 (0.82)  -0.13 (0.08) | 0.358  -1.637 | 0.722  0.111 | 0.07 (0.54)  -0.02 (0.07) | 0.134  -0.288 | 0.893  0.775 |
| **Parietal**  ***Main effect***  ***Interaction × time*** | -0.66 (0.62)  -0.07 (0.08) | -1.062  -0.837 | 0.291  0.406 | -0.17 (0.41)  -0.03 (0.06) | -0.411  -0.491 | 0.682  0.625 |
| **Sensorimotor**  ***Main effect***  ***Interaction × time*** | -0.58 (0.52)  -0.08 (0.06) | -1.130  -1.189 | 0.262  0.240 | -0.22 (0.34)  -0.06 (0.05) | -0.663  -1.230 | 0.510  0.224 |

* Indicates significant results before adjusting for multiple comparisons with FDR (q < 0.05).

**Supplementary Table 4. Comparison of linear mixed models that included the interaction between time and tau, or the interaction between time and all covariates**

|  | **Memory scores** | | | | **Executive scores** | | | |
| --- | --- | --- | --- | --- | --- | --- | --- | --- |
|  | **AIC** | **BIC** | **logLik** | **ANOVA**  **P value** | **AIC** | **BIC** | **logLik** | **ANOVA**  **P value** |
| **Medial temporal**  ***Interaction × time***  ***Interaction time × all covariates*** | 682.2  684.1 | 732.08  750.61 | -329.10  -326.05 | 0.192 | 439.84  441.77 | 489.65  508.18 | -207.92  -204.88 | 0.194 |
| **Parahippocampal**  ***Interaction × time***  ***Interaction time × all covariates*** | 681.87  685.19 | 731.75  751.70 | -328.93  -326.59 | 0.321 | 440.02  442.06 | 489.82  508.46 | -208.01  -205.03 | 0.202 |
| **Left inferior occipital**  ***Interaction × time***  ***Interaction time × all covariates*** | 681.31  684.35 | 731.19  750.86 | -328.65  -326.17 | 0.292 | 439.17  440.49 | 488.35  507.07 | -207.27  -204.33 | 0.208 |
| **Right inferior occipital**  ***Interaction × time***  ***Interaction time × all covariates*** | 684.51  686.96 | 734.39  753.47 | -330.25  -327.48 | 0.236 | 439.47  441.31 | 489.28  507.72 | -207.74  -204.66 | 0.187 |
| **Superior frontal**  ***Interaction × time***  ***Interaction time × all covariates*** | 685.68  688.09 | 735.57  754.61 | -330.84  -328.05 | 0.232 | 434.27  436.90 | 484.08  503.31 | -205.13  -202.45 | 0.251 |
| **Anterior frontal**  ***Interaction × time***  ***Interaction time × all covariates*** | 688.21  688.30 | 738.10  754.81 | -332.11  -328.15 | 0.095 | 438.71  439.58 | 488.52  505.99 | -207.36  -203.79 | 0.129 |
| **Lateral Temporo-Parietal**  ***Interaction × time***  ***Interaction time × all covariates*** | 682.54  685.93 | 732.42  752.44 | -329.27  -326.97 | 0.330 | 439.17  440.49 | 488.97  506.90 | -207.58  -204.25 | 0.154 |
| **Orbitofrontal**  ***Interaction × time***  ***Interaction time × all covariates*** | 686.35  688.58 | 736.24  755.09 | -331.18  -328.29 | 0.217 | 440.03  442.09 | 489.84  508.50 | -208.02  -205.04 | 0.203 |
| **Parietal**  ***Interaction × time***  ***Interaction time × all covariates*** | 686.97  688.50 | 736.85  755.01 | -331.48  -328.25 | 0.167 | 439.57  441.76 | 489.38  508.17 | -207.79  -204.88 | 0.213 |
| **Sensorimotor**  ***Interaction × time***  ***Interaction time × all covariates*** | 685.98  687.65 | 735.86  754.16 | -330.99  -327.83 | 0.176 | 437.6  440.0 | 487.4  440.0 | -206.8  -204.0 | 0.234 |

AIC, Akaike Information Criterion; BIC, Bayesian Information Criterion, logLik, log-likelihood; ANOVA, analysis of variance comparing the performance of each pair of models.

**Supplementary Table 5. Overlap in the number of individuals with high tau values across the spatial components**

|  | Parahippo- campal | Left Inf. Occipital | Right Inf. Occipital | Orbito- frontal | Medial Temporal | Lat. Temporo- Parietal | Parietal | Superior Frontal | Sensori- motor | Anterior Frontal |
| --- | --- | --- | --- | --- | --- | --- | --- | --- | --- | --- |
| Parahippo- campal | **29** | 13 | 11 | 11 | 11 | 4 | 2 | 1 | 1 | 1 |
| Left Inf. Occipital | - | **18** | 12 | 9 | 8 | 6 | 3 | 2 | 1 | 2 |
| Right Inf. Occipital | - | - | **14** | 6 | 7 | 5 | 3 | 3 | 2 | 2 |
| Orbito- frontal | - | - | - | **14** | 7 | 4 | 3 | 1 | 1 | 1 |
| Medial Temporal | - | - | - | - | **13** | 2 | 2 | 2 | 1 | 1 |
| Lat. Temporo- Parietal | - | - | - | - | - | **7** | 2 | 2 | 0 | 2 |
| Parietal | - | - | - | - | - | - | **6** | 3 | 1 | 1 |
| Superior Frontal | - | - | - | - | - | - | - | **4** | 1 | 1 |
| Sensori- motor | - | - | - | - | - | - | - | - | **3** | 0 |
| Anterior Frontal | - | - | - | - | - | - | - | - | - | **2** |

The table contains the number of subjects with high tau in each component (bold values) and the overlap of high tau between components. For example, the first row shows that out of the 29 subjects with high tau in the parahippocampal component, 13 had high tau in the left inferior occipital component, 11 had high tau in the right inferior occipital component and in the orbitofrontal component, 4 had high tau in the lateral temporo-parietal component, 2 had high tau in the parietal component, and 1 had high tau in the superior frontal, the sensorimotor and the anterior frontal component. Lat, lateral; Inf, inferior.
